# A Novel Diagnostic Target in the Hepatitis C Virus Genome

**DOI:** 10.1371/journal.pmed.1000031

**Published:** 2009-02-10

**Authors:** Jan Felix Drexler, Bernd Kupfer, Nadine Petersen, Rejane Maria Tommasini Grotto, Silvia Maria Corvino Rodrigues, Klaus Grywna, Marcus Panning, Augustina Annan, Giovanni Faria Silva, Jill Douglas, Evelyn S. C Koay, Heidi Smuts, Eduardo M Netto, Peter Simmonds, Maria Inês de Moura Campos Pardini, W. Kurt Roth, Christian Drosten

**Affiliations:** 1 Clinical Virology Group, Bernhard Nocht Institute for Tropical Medicine, Hamburg, Germany; 2 Institute of Virology, University of Bonn, Bonn, Germany; 3 Infectious Diseases Research Laboratory, University Hospital Prof. Edgard Santos, Federal University of Bahia, Salvador, Brazil; 4 University of São Paulo State (UNESP), Botucatu Medical School, Blood Transfusion Centre - Molecular Biology Laboratory and Internal Medicine Department, Botucatu, São Paulo, Brazil; 5 Virus Evolution Group, Centre for Infectious Diseases, University of Edinburgh, Edinburgh, United Kingdom; 6 Department of Pathology, Yong Loo Lin School of Medicine, National University of Singapore; 7 Molecular Diagnosis Centre, National University Hospital, Singapore; 8 Division Medical Virology/National Health Laboratory Service, Department of Clinical Laboratory Sciences, Faculty of Health Sciences, University of Cape Town, Cape Town, South Africa; 9 GFE Blut mbH, Frankfurt (Main), Germany; Oxford University, United Kingdom

## Abstract

**Background:**

Detection and quantification of hepatitis C virus (HCV) RNA is integral to diagnostic and therapeutic regimens. All molecular assays target the viral 5′-noncoding region (5′-NCR), and all show genotype-dependent variation of sensitivities and viral load results. Non-western HCV genotypes have been under-represented in evaluation studies. An alternative diagnostic target region within the HCV genome could facilitate a new generation of assays.

**Methods and Findings:**

In this study we determined by de novo sequencing that the 3′-X-tail element, characterized significantly later than the rest of the genome, is highly conserved across genotypes. To prove its clinical utility as a molecular diagnostic target, a prototype qualitative and quantitative test was developed and evaluated multicentrically on a large and complete panel of 725 clinical plasma samples, covering HCV genotypes 1–6, from four continents (Germany, UK, Brazil, South Africa, Singapore). To our knowledge, this is the most diversified and comprehensive panel of clinical and genotype specimens used in HCV nucleic acid testing (NAT) validation to date. The lower limit of detection (LOD) was 18.4 IU/ml (95% confidence interval, 15.3–24.1 IU/ml), suggesting applicability in donor blood screening. The upper LOD exceeded 10^−9^ IU/ml, facilitating viral load monitoring within a wide dynamic range. In 598 genotyped samples, quantified by Bayer VERSANT 3.0 branched DNA (bDNA), X-tail-based viral loads were highly concordant with bDNA for all genotypes. Correlation coefficients between bDNA and X-tail NAT, for genotypes 1–6, were: 0.92, 0.85, 0.95, 0.91, 0.95, and 0.96, respectively; X-tail-based viral loads deviated by more than 0.5 log10 from 5′-NCR-based viral loads in only 12% of samples (maximum deviation, 0.85 log10). The successful introduction of X-tail NAT in a Brazilian laboratory confirmed the practical stability and robustness of the X-tail-based protocol. The assay was implemented at low reaction costs (US$8.70 per sample), short turnover times (2.5 h for up to 96 samples), and without technical difficulties.

**Conclusion:**

This study indicates a way to fundamentally improve HCV viral load monitoring and infection screening. Our prototype assay can serve as a template for a new generation of viral load assays. Additionally, to our knowledge this study provides the first open protocol to permit industry-grade HCV detection and quantification in resource-limited settings.

## Abstract 2

Christian Drosten and colleagues develop, validate, and make openly available a prototype hepatitis C virus assay based on the conserved 3' X-tail element, with potential for clinical use in developing countries.

## Editors' Summary

**Background.:**

About 3% of the world's population (170 million people) harbor long-term (chronic) infections with the hepatitis C virus (HCV) and about 3–4 million people are newly infected with this virus every year. HCV—a leading cause of chronic hepatitis (inflammation of the liver)—is spread through contact with the blood of an infected person. Globally, the main routes of transmission are the use of unscreened blood for transfusions and the reuse of inadequately sterilized medical instruments, including needles. In affluent countries, where donated blood is routinely screened for the presence of HCV, most transmission is through needle sharing among drug users. The risk of sexual and mother-to-child transmission of HCV is low. Although HCV infection occasionally causes an acute (short-lived) illness characterized by tiredness and jaundice (yellow eyes and skin), most newly infected people progress to a symptom-free, chronic infection that can eventually cause liver cirrhosis (scarring) and liver cancer. HCV infections can be treated with a combination of two drugs called interferon and ribavirin, but these drugs are expensive and are ineffective in many patients.

**Why Was This Study Done?:**

An effective way to limit the global spread of HCV might be to introduce routine screening of the blood that is used for transfusions in developing countries. In developed countries, HCV screening of blood donors use expensive, commercial “RT-PCR” assays to detect small amounts of HCV ribonucleic acid (RNA; HCV stores the information it needs to replicate itself—its genome—as a sequence of “ribonucleotides”). All the current HCV assays, which can also quantify the amount of viral RNA in the blood (the viral load) during treatment, detect a target sequence in the viral genome called the 5′-noncoding region (5′-NCR). However, there are several different HCV “genotypes” (strains). These genotypes vary in their geographical distribution and, even though the 5′-NCR sequence is very similar (highly conserved) in the common genotypes (HCV genotypes 1–6), the existing assays do not detect all the variants equally well. This shortcoming, together with their high cost, means that 5′-NCR RT-PCR assays are not ideal for use in many developing countries. In this study, the researchers identify an alternative diagnostic target sequence in the HCV genome—the 3′-X-tail element—and ask whether this sequence can be used to develop a new generation of tests for HCV infection that might be more appropriate for use in developing countries.

**What Did the Researchers Do and Find?:**

The researchers determined the RNA sequence of the 3′-X-tail element in reference samples of the major HCV genotypes and showed that this region of the HCV genome is as highly conserved as the 5′-NCR. They then developed a prototype X-tail RT-PCR assay and tested its ability to detect small amounts of HCV and to measure viral load in genotype reference samples and in a large panel of HCV-infected blood samples collected in Germany, the UK, Brazil, South Africa, and Singapore. The new assay detected low levels of HCV RNA in all of the genotype reference samples and was also able to quantify high RNA concentrations. The viral load estimates it provided for the clinical samples agreed well with those obtained using a commercial assay irrespective of the sample's HCV genotype. Finally, the X-tail RT-PCR assay gave similar results to a standard assay at a fraction of the cost when used to measure viral loads in a Brazilian laboratory in an independent group of 127 patient samples collected in Brazil.

**What Do These Findings Mean?:**

These findings suggest that the HCV 3′-X-tail element could provide an alternative target for screening blood samples for HCV infection and for monitoring viral loads during treatment, irrespective of HCV genotype. In addition, they suggest that X-tail RT-PCR assays may be stable and robust enough for use in laboratories in emerging countries. Overall, these findings should stimulate the development of a new generation of clinical HCV assays that, because the protocol used in the X-tail assay is freely available, could improve blood safety in developing countries by providing a cheap and effective alternative to existing proprietary HCV assays.

**Additional Information.:**

Please access these Web sites via the online version of this summary at http://dx.doi.org/10.1371/journal.pmed.1000031.

- The World Health Organization has a fact sheet about hepatitis C (in English and French)
- The US Centers for Disease Control and Prevention provides information on hepatitis C for the public and for health professionals (information is also available in Spanish)
- The US National Institute of Diabetes and Digestive and Kidney Diseases provides basic information on hepatitis C (in English and Spanish)
- The MedlinePlus Encyclopedia has a page on hepatitis C; MedlinePlus also provides links to further information on hepatitis C (in English and Spanish)

## Introduction

Hepatitis C virus (HCV) is one of the leading causes of chronic hepatitis, liver cirrhosis, and hepatocellular carcinoma [1,2]. Seroprevalence studies suggest that at least 170 million individuals have been infected worldwide [3]. The incidence of new HCV infections has decreased in affluent countries owing to screening of blood products, but an increase of global patient numbers is still expected [1–3]. At present, six genotypes and more than 30 subtypes are well characterized, with an overall nucleotide diversity of 31%–33% between genotypes and 20%–25% between subtypes [4]. Diagnostic detection of HCV RNA identifies an infection weeks to months before a detectable antibody response. To prevent transmission by transfusion of viremic blood, nucleic acid testing (NAT) of blood donors has become a routine procedure in industrialized countries. As a marker of treatment success, the decrease of virus RNA concentration (viral load) as measured by quantitative NAT has become a clinical gold standard [5,6]. For these molecular tests, several generations of qualitative and/or quantitative HCV NAT assays have been in use. The most recent improvement was the introduction of real-time PCR-based assays [7–10]. However, even the latest versions of assays diverge in performance between different genotypes [11–21]. This divergence is of strategic clinical relevance as success of therapy varies between HCV genotypes [22]. Consistently, genotypes other than those occurring in industrialized countries have been under-represented in clinical evaluation studies [23]. Along with high costs for commercial assays, genotype variation complicates the initiation of successful treatment programmes in several less-affluent countries. In analogy to HIV treatment, open-protocol and low-cost HCV viral load technology would be desirable [24–28].

Unfortunately, the design of both commercial and in-house tests for HCV suffers from properties of the viral 5′-noncoding region (5′-NCR). This region is thought to be the most conserved portion of the HCV genome [29] and is targeted by all assays. Test manufacturers have made substantial efforts to optimize 5′-NCR target sites, keeping them unpublished for all current commercial assays. Still, there seem to be remaining issues with these tests, as exemplified by the discontinuation of a new real-time PCR-based assay in 2004 after initial introduction in blood screening [13,30]. One of the most important issues may be the existence of complex secondary structures due to the 5′-NCR's function as an internal ribosomal entry site for genome translation [31]. These secondary structures may interfere in complex ways with primer or probe binding.

Due to notorious problems with the 5′-NCR, a different target region would be beneficial. However, viral genes downstream of the 5′-NCR are not useful as diagnostic targets because of their nucleotide variability [4,32]. In the mid 1990s, molecular biology studies on the HCV replication cycle revealed that the genome carries an additional element in its 3′-UTR downstream of the poly-U tract, the so-called X-tail [33,34]. The biological function of the X-tail is not completely understood, but it has been shown that it is involved in several crucial steps throughout the HCV life cycle, involving both RNA-RNA and RNA-protein interactions [35–38]. The X-tail has been suggested to be highly conserved [35,36], but it is unclear whether other issues (e.g., secondary structures) might interfere with its molecular detection. It may be due to lack of available sequence information across all genotypes that the X-tail has been neglected as a diagnostic target so far. In this study we explored its utility for HCV detection and quantification by sequencing the X-tail from a broad and complete panel of HCV genotypes. To assess its diagnostic utility, we then developed an X-tail-based viral load assay that fulfills the same technical standards as commercial assays currently in clinical use. Clinical validation involved 725 stored samples across HCV genotypes 1–6 from patients from four continents (Germany, UK, South Africa, Singapore, and Brazil). Technical robustness was demonstrated by implementation of the assay in a routine viral load laboratory in Brazil.

## Materials and Methods

### Reference Plasma

The World Health Organization (WHO) international standard reagent 96/798 was obtained from National Institute for Biological Standards and Control, (Hertfordshire, UK) [39]. It contained 50,000 international units (IU) of HCV, genotype 1a, per milliliter. A genotype reference plasma panel was obtained from the German Hepatitis C Reference Center at the University of Essen, including lyophilized human plasma containing HCV genotypes 1a, 1b, 2a, 2b, 2c, 2i, 3a, 4d, 5a, and 6e. Genotypes as determined by commercial assays (Inno-Lipa HCV2, GEN-ETI-K DEIA) and in-house sequencing, as well as viral loads as determined by both the Roche Amplicor 2.0 (Amplicor) and the Bayer VERSANT bDNA 3.0 (branched DNA [bDNA]) systems were provided with this panel (http://www.uni-essen.de/virologie/hc_hcv-panel.html). Only the samples mentioned in this paragraph were used for the technical development of the assay.

### Patient Plasma and Commercially Available Viral Load and Genotyping Assays

For validation of the assay, a total of 725 human plasma samples (one sample per patient) were studied. Of these, 598 were obtained from routine clinical testing in Germany, UK, South Africa, Singapore, and Brazil (*n* = 319, 62, 102, 52, 46, and 17 of HCV genotypes 1, 2, 3, 4, 5, and 6, respectively). These stored samples had been selected because of their known genotypes, and were collected at the University of Bonn, Germany (*n* = 530, HCV genotypes 1 to 4, sampled from 2003 to 2007), the University of Cape Town, South Africa (*n* = 46, genotype 5, sampled from 1996 to 2007), the National University of Singapore (*n* = 9, genotypes 3k and 6, sampled from 2006 to 2007), and the University of Edinburgh, UK (*n* = 13, genotype 6, sampled from 1999 to 2004). Samples were generally taken at the time of genotyping, i.e., immediately before the beginning of therapy. We did not accept recorded genotype information from earlier or later samples from a given patient. An additional set of plasma samples, collected from 2005 to 2007 at the University of São Paulo State, Brazil, contained 127 nongenotyped samples from patients during HCV therapy. All samples were anonymized and approval was obtained by the ethical committees of the University of Bonn Medical Centre, Bonn, Germany; the University of São Paulo State (UNESP); Botucatu Medical School, Botucatu, São Paulo, Brazil; and the Federal University of Bahia Medical School, Salvador, Bahia, Brazil. Viral loads in all samples were determined at the University of Bonn with bDNA on a VERSANT 440 Molecular System. Samples were genotyped by the VERSANT HCV Genotype assay (Line Probe assay [LiPA]) (Bayer Diagnostics). Genotyping of the South African and British samples was done by restriction fragment length polymorphism (RFLP), as previously described [40,41] and confirmed by LiPA. The genotypes of all samples from Singapore were confirmed by sequencing of the 5′-NCR and nonstructural protein 5b (NS5b) domains [42]. Samples were stored at −20 °C until quantification with the X-tail real-time reverse transcriptase (RT)-PCR assay (X-tail RT-PCR). All Brazilian samples were tested by bDNA on a VERSANT 340 Molecular System and X-tail RT-PCR in the local laboratory in Brazil.

### Quantification of HCV Viral Load by X-Tail RT-PCR

Reactions of 50 μl contained 20 μl of RNA extract, 5× reaction buffer (Qiagen One-step RT-PCR kit), 200 μM of each dNTP, 200 nM of primer XTF5 (GTGGCTCCATCTTAGCCCTAGT), 300 nM of primer HCMgR2 (TGCGGCTCACGGACCTTT), 100 nM of HCV-specific probe HCVMGB2 (FAM-CACGGCTAGCTGTG-Black Hole Quencher 2/Minor grove binder), 100 nM of Internal Control-probe YFPY (VIC-ATCGTTCGTTGAGCGATTAGCAG-Black Hole Quencher 1), and 2 μl of Enzyme Mix. Thermal cycling was done on Applied Biosystems 7700 or 7500 SDS instruments under the following conditions: 55 °C, 10 min; 95 °C, 15 min; 45 cycles at 94 °C, 10 s and 58 °C, 15 s.

### Computing

Statistical analyses were performed with the SPSS 13 (SPSS). Alignments were generated using BioEdit (http://www.mbio.ncsu.edu/BioEdit/bioedit.html). Sequences were analyzed using the Lasergene software package (DNAStar). All database work was performed online on Los Alamos National Laboratory (LANL) (http://hcv.lanl.gov/content/hcv-index) and euHCV (http://euhcvdb.ibcp.fr/euHCVdb) domains.

### Supplemental Materials and Methods

A STARD diagram and checklist are included in Figure S1 and Text S1. A more detailed description of the X-tail NAT assay including protocols for RNA extraction and data on technical performance are provided in Text S2. A bench protocol and instructions for requesting controls is provided in Text S3.

### Accession Numbers

Sequences determined in this study have been submitted to GenBank (http://www.ncbi.nlm.nih.gov/Genbank) under accession numbers EU835523-EU835532.

## Results

### Analysis of the X-Tail Region

**Figure 1:**
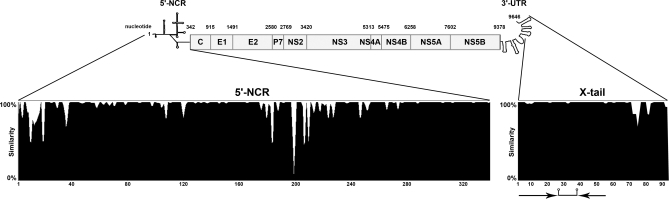
Nucleotide Similarities within HCV 5′- and 3′-Genome Ends Top panel: schematic representation of the HCV reference genome H77 as given in the 2008 LANL database. Bottom panel: Percent nucleotide identity along the 5′-NCR and the X-tail, as calculated by a sliding window analysis with VectorNTI software. Window size was 2 nucleotides. The alignment used for the sliding window analysis contained the complete HCV genotype reference panel (*n* = 60 sequences) from the LANL HCV database (available at http://hcv.lanl.gov/content/sequence/NEWALIGN/align.html) and all X-tail sequences available in GenBank as shown in Text S2, including the sequences determined de novo in this study (EU835523-EU835532).

In order to examine the conservedness of the X-tail region, a nucleic acid sequence alignment was generated that contained all X-tail sequences stored in the LANL and euHCV databases as of January 2007. These databases comprised sequences classified as genotypes 1, 2, and 3. Sets of sequencing primers for the ultimate 3′-end of the genome were identified, and the X-tail was sequenced from reference plasma samples of HCV genotypes 1–6. Together with one outlier sequence taken from the initial characterization study on the X-tail [33] that was found later on to correspond to genotype 4 (PS, unpublished data), these novel sequences were added to the alignment. As shown in Figure 1, the degree of conservedness in the X-tail was comparable to that of the 5′-NCR, which is the target of all existing viral load assays.

### Experimental Identification of a Diagnostic Target Region within the X-Tail

The complete 3′-UTR alignment was evaluated as a target for real-time PCR design. Since primer design software was not able to identify suitable candidate oligonucleotides, in a first approach five forward primers, five reverse primers, and two probes were selected upon inspection of the alignment. All of the 50 resulting real-time PCR sets were tested experimentally for reaction efficiency. However, even after selection of the most effective oligonucleotides and optimization of reaction conditions, the lower limit of detection (LOD) did not fall below 150 IU/ml (see Text S2 for additional information on technical evaluation procedures). Since primers had a perfect match with the target sequence, the limitation in sensitivity was ascribed to RNA secondary structures possibly interfering with oligonucleotide hybridization.

**Figure 2:**
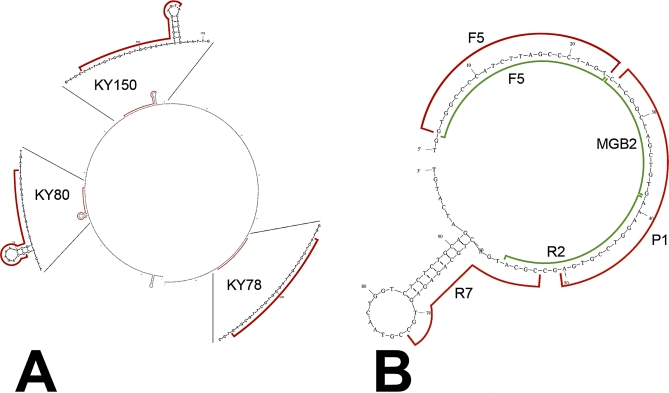
Secondary Structure Predictions Secondary structure of the HCV 5′-NCR (A) and the 3′-X-tail (B) of HCV 1a reference genome H77 as predicted by MFOLD [43]. Prediction was done at 50 mM salt concentration and PCR primer annealing temperature (58 °C). Free energies ΔG were −1.6 × 10^−3^ J/mol for (A) and −5,06 × 10^−4^ J/mol for (B). 5′-NCR structure predictions were identical for all genotypes in the LANL genotype reference panel except genotype 2, which had one additional stem-loop element (not covered by primers, not shown in [A]). Structure predictions for all X-tail sequences (database and new sequences) were identical (not shown in [B]). (A) Binding sites of oligonucleotides used by the Roche Amplicor assay are shown in red (sense primer KY80, hybridization probe KY150, antisense primer KY78). Nucleotide variability at these binding sites is shown in Text S2). (B) Outer lines in red identify the hybridization sites of oligonucleotides used in initial X-tail RT-PCR set, yielding limited sensitivity (forward primer F5, TaqMan probe P1, reverse primer R7). Interior lines in green identify the final X-tail prototype assay (forward primer F5, reverse primer R2, probe MGB2). Nucleotide variability at these sites is shown in Text S2.

RNA secondary structures of both the X-tail and the 5′-NCR were modeled at PCR primer annealing conditions (58 °C and 50 mM ion concentration) using MFOLD [43]. In the 5′-NCR, secondary structures in all genotypes were almost completely dissolved, but still a few stable stem-loop elements remained. Of note, some of these fell in the conserved regions targeted by a prototypic 5′-NCR assay, the Roche Amplicor Monitor, and probably also the later generation COBAS TaqMan system [13]. For the X-tail, a 10-nucleotide-stem element in the stem-loop 1 region towards the 3′ end of the X-tail was predicted to be stable at the same conditions. Figure 2 shows the structure predictions with exemplified oligonucleotides for X-tail and 5′-NCR. It was concluded that stable stem structures were likely to interfere with binding of all antisense primers and most of the probes used up to this point.

### Prototype Viral Load Assay Based on the HCV X-Tail

Consequently, the antisense primer and probe binding sites were moved further upstream into the amplicon. Out of several new candidate primers and probes, an oligonucleotide combination was determined that provided very high amplification efficiency (final assay oligonucleotides as shown in the Materials and Methods section). These primers and probe were directly adjacent, without unoccupied nucleotides between them. Very few nucleotide mismatches occurred with any of the sequences taken from GenBank or determined from reference plasma (see Figure 2). According to established data, these mismatches were highly unlikely to interfere with assay performance [44–46].

To assess the diagnostic applicability of the X-tail, this PCR set was developed into a clinical-grade prototype assay. The input RNA volume was maximized to 20 μl in a 50-μl assay volume, and the chemistry was adjusted for use with a semi-automated nucleic acids extraction. In order to detect PCR inhibition, a competitive internal control was incorporated. It was amplified by the same primers as the diagnostic target but was detected by a probe of different sequence composition and different fluorescent labeling. To enhance assay stability, control RNA was formulated to be nuclease resistant and added at working concentration to all reactions at the lysis buffer stage. As a reference standard for viral loads, a noninfectious and stable copy of the HCV 1a X-tail was cloned in an armored RNA phage (refer to Text S2 for synthesis and calibration of internal control and reference standard).

Before evaluation of its technical and clinical performance, the specificity of the assay for HCV was confirmed on cell culture supernatants or sera containing the following *Flaviviridae* species: dengue virus 1, 2, 3, and 4; yellow fever virus; tick-borne encephalitis virus; St. Louis encephalitis virus; West Nile virus; and hepatitis G virus (HCV)/GB virus-C. Due to frequent co-infection of patients with HIV-1 and HCV, an HIV-1 genotype reference panel comprising 11 HIV-1 genotypes from groups M, N, and O [47] was acquired from National Institute for Biological Standards and Control (product code 01/466) and tested with X-tail RT-PCR. High RNA content in all of these materials was confirmed by a flavivirus genus-specific RT-PCR and an HIV-1 in-house real time RT-PCR assay [28,48]. No nonspecific amplification occurred with any of these materials (unpublished data).

### LOD and Quantification Range

**Figure 3:**
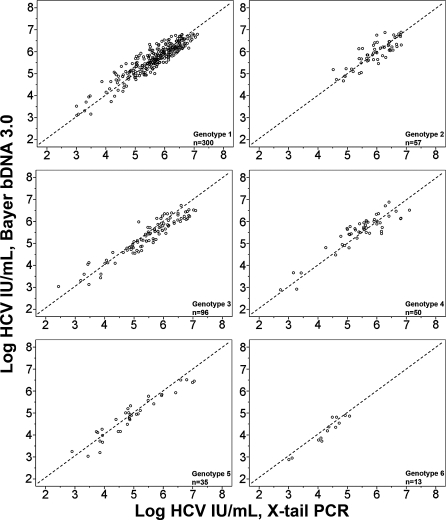
Correlation of Viral Loads as Determined by X-Tail RT-PCR (*x*-Axis) and bDNA (*y*-Axis) Genotypes and numbers of samples (*n*) are given in the bottom right corner of each panel. Pearson's bivariate correlation coefficients were 0.92, 0.85, 0.95, 0.91, 0.95, and 0.96 for HCV genotypes 1 to 6, respectively. The dashed lines represent ideal correlations. Genotype 5, 0.07/0.31; and genotype 6, 0.12/0.19.

A common technical specification for the sensitivity of NAT is the 95% LOD, i.e., the concentration down to which >95% of iterative tests will detect virus. 95% LODs for the X-tail-based prototype assay were determined for each individual genotype by parallel limiting dilution testing as described in Text S2. Results indicated high sensitivity across all genotypes (Table 1). The overall LOD was as low as 18.4 IU/ml (Figure 3).

**Table 1:**
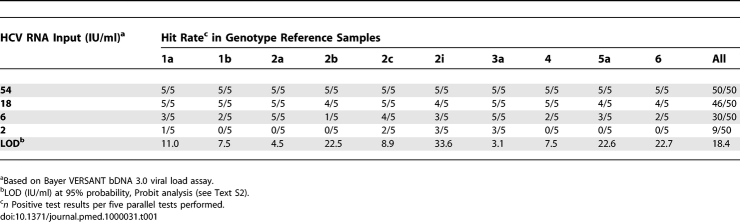
Lower LOD by HCV Genotype

Next, the upper quantification limit was evaluated, i.e., the maximal RNA concentration that can be measured without technical bias contributed by the system. This is relevant because HCV patients may show extraordinarily high viral loads. Synthetic Armored RNA standards exceeding clinically observed viral loads (1,890,000, 18,900,000, and 189,000,000 IU/ml) were tested. The determined quantities did not deviate from those expected by dilution factor (unpublished data).

### Accuracy in Viral Load Monitoring

Intra-assay coefficient of variation (CV) at 7,245,000 IU/ml of HCV RNA was 5.76% with a standard deviation (SD) of 0.03 log10. Inter-assay CVs ranged from 6.11 to 18.42% (SDs, 0.03–0.09 log10) at 1,890–189,000 IU/ml (refer to Text S2).

To evaluate the utility of the X-tail in viral load monitoring, the prototype assay was compared against bDNA. The latter was chosen as a clinical gold standard because it represented a well-established assay that is more robust against genotype bias than other clinical assays [49–51]. All of the 598 plasma samples, covering HCV genotypes 1–6 and all ranges of viral loads occurring in HCV patients, were tested with X-tail RT-PCR and results compared to bDNA. Samples with results below or above the quantification ranges of bDNA or X-tail RT-PCR (*n* = 47) were excluded from correlation analysis, leaving 551 samples. As shown in Figure 3, X-tail RT-PCR correlated well with bDNA for all genotypes tested, with correlation coefficients of 0.92, 0.85, 0.95, 0.91, 0.95, and 0.96 for HCV genotypes 1 to 6, respectively. All correlations were highly significant (*p* < 0.01 for all, Pearson's goodness of fit test).

**Figure 4:**
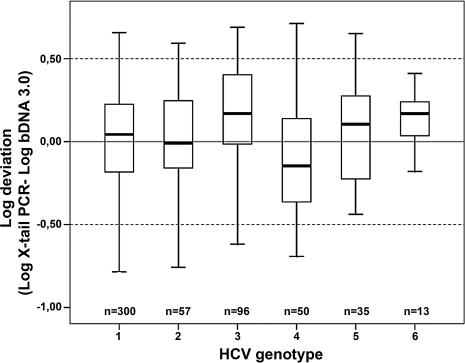
Quantitative Deviations between X-Tail–Based and bDNA Viral Loads, per Genotype (*y*-Axis) Genotypes are indicated below the *x*-axis, the number of samples tested per genotype (*n*) above the *x*-axis. Each box shows the median, interquartile range (box length, containing 50% of data) and whiskers show extreme values (there were no statistical outliers). Deviations between X-tail RT-PCR and bDNA [log10 X-tail RT-PCR − log10 bDNA] were genotype 1, 0.00/0.31 (mean/SD); genotype 2, 0.01/0.33; genotype 3, 0.17/0.32; genotype 4, −0.09/0.37; genotype 5, 0.07/0.31; and genotype 6, 0.12/0.19.

Figure 4 shows differences in absolute quantification results for genotypes 1–6. Differences of more than 0.5 log10 occurred infrequently (12.0% of samples in total; maximally observed deviation: 0.85 log10), with a balanced distribution to both higher and lower viral loads (5.1% and 6.9%, respectively). Mean and median log10 differences and SDs were small for all six genotypes, and all were below clinical significance (Figure 4). X-tail RT-PCR yielded minimally higher viral loads than bDNA for all genotypes except genotype 4 (mean log10 differences were 0.00, 0.01, 0.17, −0.09, 0.07, and 0.12 for genotypes 1 to 6, respectively). In order to assess whether the slight underquantification of genotype 4 could have been caused by a systematic error, the two samples that showed strongest underquantification (up to 0.7 log10) were sequenced. They showed no nucleotide mismatches at the oligonucleotide binding sites, suggesting other reasons for the observed deviations (handling, storage, error in the gold standard, etc.).

### Clinical Sensitivity

Because correlation analysis only included samples that were positive in both tests, it did not reflect overall clinical sensitivity. A comparison of qualitative detection rates is shown in Table 2. Sixteen of a total of 598 samples (2.68%) were below the detection limit of bDNA but detectable by X-tail RT-PCR at a median viral load of 284 IU/ml. Three of these samples had viral loads that should have been detectable with bDNA, according to its LOD (615 IU/ml). Two samples were negative by X-tail RT-PCR despite RNA detection by bDNA at viral loads of 989 and 4,681 IU/ml, respectively.

**Table 2:**
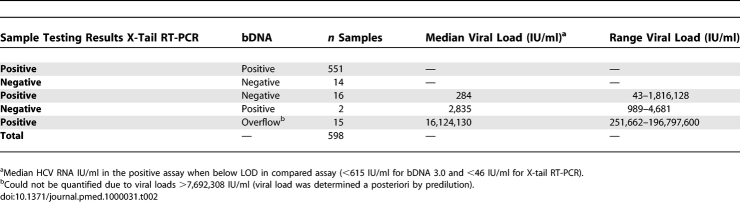
Detection of HCV by X-Tail RT-PCR Versus bDNA

A total of 15 samples had viral loads above the upper cut-off of bDNA, requiring predilution and repetition of the bDNA assay. All of them were quantifiable by X-tail RT-PCR upon first testing.

### Potential for Implementation in Resource-Limited Settings

**Figure 5:**
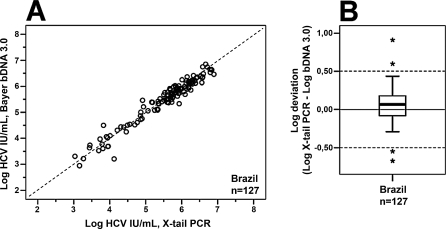
Results Obtained with X-Tail Viral Load Monitoring, Implemented in Brazil 127 samples were measured in a Brazilian HIV-1 viral load monitoring centre by Bayer VERSANT 3.0 HCV bDNA assay and by X-tail in-house PCR. (A) Correlation of viral loads between X-tail RT-PCR (*x*-axis) and Bayer bDNA 3.0 (*y*-axis). The dashed line represents an ideal correlation. Pearson's bivariate correlation coefficient was 0.97. (B) Quantitative differences. The box shows the median and interquartile range (box length). The whiskers represent an extension of the 25th or 75th percentiles by 1.5 times the interquartile range. Datum points beyond the whisker range are considered as outliers and marked as asterisks.

Affordable viral load monitoring would be desirable in resource-limited settings with high HCV prevalence. To evaluate whether the X-tail prototype assay would provide adequate stability and quality in comparison to 5′-NCR-based assays, it was implemented in a diagnostic center involved in the Brazilian HCV treatment program. Protocols, controls, and standards for X-tail NAT were provided to the laboratory in Brazil. All other reagents were purchased locally. Plasma samples from 127 patients were tested by bDNA and X-tail NAT. The correlation coefficient between viral loads obtained with both assays was 0.97 (*p* < 0.001) at a mean quantitative difference of 0.05 log10 (SD 0.018). As shown in Figure 5, almost all quantitative differences were below 0.5 log10. Only four outlier samples occurred, with deviations to higher and lower quantification results of up to 1 log10 in X-tail RT-PCR.

## Discussion

In this study we have identified a new diagnostic target region in the HCV genome. A prototype RT-PCR assay based on the HCV X-tail proved to be highly sensitive (18.4 IU/ml, 95% probit probability), robust against genotype variation, and highly precise in virus quantification. The assay was efficiently implemented and projected to be highly cost efficient in an emerging country setting. These data may assist in translating state-of-the art diagnostic technology to less affluent settings.

The X-tail region of the HCV genome has been known for several years [33,34] but has not been used as a diagnostic target so far. Its biological functions are now becoming clearer, and it is likely that its role in the virus life cycle involves both RNA and protein interactions. The three X-tail stem-loop structures (SL1–3) seem to be involved in negative-strand synthesis and significantly enhance HCV replication by means of binding to ribosomal and other cellular proteins [35–38]. A kissing-loop interaction between an X-tail stem-loop structure (SL2) and an RNA loop within the nonstructural protein 5b (NS5b) region was shown to be essential for HCV replication [52]. These multiple mechanisms of interaction imply a high degree of conservedness in the X-tail, which could indeed be confirmed for all genotypes in this study. As anticipated [32,53], the region proved as conserved as the 5′-NCR across all genotypes, and seemed to exhibit less problematic structural features than the 5′-NCR.

To circumvent the limitations that the 5′-NCR presents in diagnostics, we have established the HCV X-tail as a target for molecular detection and quantification. Initially we could not be sure about its utility for several reasons implied by its biological functions. A possible limitation could have been its position at the end of the genome and beyond the poly-U tract, making the X-tail prone to nuclease degradation. Moreover, due to its functions as a 3′-element it could have been associated strongly with viral or cellular proteins. Although we could not investigate these issues in our study, we are confident about its diagnostic utility from the clinical part of our study, showing that X-tail-based viral loads were highly concordant with results from bDNA testing. bDNA was chosen as a gold standard because it uses multiple probes along the 5′-NCR and initial core region and has proven to be the most robust viral load test compared to other assays [49–51]. We used a large panel of clinical specimens that included sufficient numbers of samples of genotypes 1–6 (*n* = 725 in total, 598 genotyped samples). To our knowledge, this is the most diversified and comprehensive panel of clinical specimens used in the validation of HCV NAT so far [7,10,14,17–21,49,54,55]. For genotypes 4–6, earlier studies relied on sparse genotype reference samples, which have also been used for the original design of assays and may under-represent the genetic diversity observed in clinical samples. Our study included field clinical samples of all genotypes in substantial numbers, sampled in four different geographic locations. The genotype-related robustness of the prototype X-tail assay was at least equivalent with that of proprietary assays [12,13,15,17–21,23,49,51,56,57]. Quantitative correlation and lower limits of detection were highly consistent for all genotypes by X-tail RT-PCR. The lower LOD (18.4 IU/ml) and the upper quantification range of our assay (>189,000,000 IU/ml) were equivalent to that of the last generation Roche COBAS TaqMan HCV test and the Abbott HCV RealTime assay [7–10,23,58]. Critically, X-tail-based viral loads and bDNA results were highly congruent, making the assay compatible with other quantification systems. This compatibility is critical when patients switch their treating institution, which may entail switching between viral load tests. The observed quantitative deviations were generally <0.5 log10, i.e., below clinical relevance.

In resource-limited settings our prototype assay could be used instead of more costly proprietary assays in HCV treatment and testing. It is not patented, has a simple and accessible formulation (refer to the bench protocol and instructions for requesting controls in Text S3), and is appropriate for rare HCV genotypes. Several less-affluent countries have established successful HIV treatment programs, but suffer from considerable HCV prevalence as well. One important example is Brazil, where networks of well-managed public laboratories conduct viral load testing as a part of the national HIV-1 treatment program [59]. Intensive efforts have been made to develop low-cost viral load assays for HIV-1 in Brazil and elsewhere [24–28]. The most important issue with such in-house assays is their robustness. In proprietary commercial assays robustness is contributed by advanced features like automated RNA preparation, nuclease resistant calibrators, and synthetic internal controls. We have incorporated all of these features in the open protocol HCV X-tail assay. A second issue in using in-house assays is quality control, which is shifted largely to the production process in commercial kits, but can be managed at the laboratory level if appropriate quality control procedures are in place. In the context of HIV viral load monitoring, we and others have demonstrated that in-house testing in emerging countries is feasible in well-managed laboratories [24,25,27,28]. The potential for translation of the assay was demonstrated by implementation in a Brazilian laboratory and on-site comparison against the Bayer bDNA assay. X-tail RT-PCR had short turnover times (2.5 h for up to 96 samples) and was implemented from provided protocols without technical difficulties. Overall results suggest that X-tail RT-PCR is suitable for the Brazilian setting, even though genotyping was not feasible at the study site. The overall HCV genotype distribution in São Paulo state is known to be approximately 65%, 5%, and 30% of genotypes 1, 2, and 3, respectively [60]. Reaction costs of the assay ranged around US$8.70 per sample, or 8.1% of the current viral load costs in Brazil. It should be mentioned that under European conditions, an additional service license fee would apply, increasing the cost of one assay to US$18 per sample (refer to Table 3 for an estimation of costs).

**Table 3:**
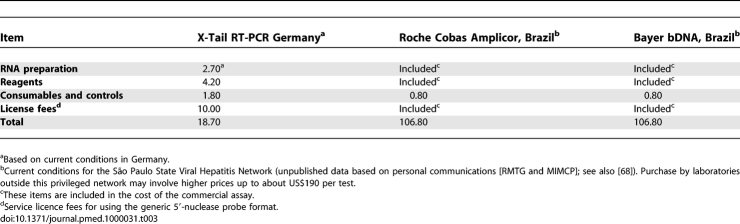
Approximate Pricing of HCV Viral Load Assays, US Dollars, without Taxes

Finally, HCV is an important issue for blood safety in emerging countries. Again, Brazil provides an example representative of many other emerging countries. An urgent federal decree in 2003 demanded the general testing of donated blood for HCV by NAT [61], but this strategy has not yet been implemented. According to estimates by the Brazilian Ministry of Health, it would cost about US$40 million to screen 4 million blood donations annually [62].

We previously demonstrated the feasibility of in-house testing of blood donors for HCV by NAT methods [63]. Due to the high sensitivity of X-tail RT-PCR (18.4 IU/ml), the prototype assay could be used for testing pooled blood donor samples. With the well-established approach of pooling 24 donors prior to NAT testing [64], the projected LOD would be 441 IU/ml per donor (18.4 IU/ml × 24). This projection would match the sensitivity achieved with pooled blood donor screening in Europe [65,66] and North America [64,67]. Calculating the cost for one X-tail NAT assay at US$10 (US$8.70 for RT-PCR according to Table 3, plus US$1.30 for extra consumables due to pooling), and assuming 20% additional costs for controls and confirmatory testing, 24-donor pool testing by X-tail RT-PCR would amount to US$2 million per 4 million donations annually (4 million/24 × US$10 × 1.2). This means a reduction down to 5% of the current economic estimate. In view of the technical robustness and transferability of the assay described in this report, we believe that such a strategy could be a realistic approach in emerging countries aiming to increase blood safety through the introduction of HCV NAT.

## Supporting Information

Alternative Language Abstract S1 Translation of the Abstract into French by Felix Drexler (29 KB DOC)

Alternative Language Abstract S2 Translation of the Abstract into German by Felix Drexler and Christian Drosten (29 KB DOC)

Alternative Language Abstract S3 Translation of the Abstract into Portuguese by Felix Drexler and Maria Inez Pardini (31 KB DOC)

Alternative Language Abstract S4 Translation of the Abstract into Spanish by Oscar Martinez Martinez (27 KB DOC)

Figure S1 STARD Diagram (11 KB PDF)

Text S1 STARD Checklist (18 KB PDF)

Text S2 Detailed Description of the X-tail NAT Assay (696 KB DOC)

Text S3 Bench Protocol and Instructions for Requesting Controls (86 KB PDF)

## Abbreviations

bDNA: branched DNA
HCV: hepatitis C virus
IU: international units
LANL: Los Alamos National Laboratory
LOD: limit of detection
NAT: nucleic acid testing
NCR: noncoding region
RT-PCR: reverse transcriptase PCR
SD: standard deviation
X-tail RT-PCR: X-tail real-time RT-PCR assay
