# Supplementary material for: A Novel Diagnostic Target in the Hepatitis C Virus Genome
Source: PLoS Med. 2009 Feb 10;6(2):e1000031. doi: 10.1371/journal.pmed.1000031 (PMC2637920; doi:10.1371/journal.pmed.1000031)
Supplement: Supplementary file 2 [file pmed.1000031.sd007.pdf]

HCV X-Tail real time RT-PCR bench protocol

MasterMix:	µL
H2O	12.5
5X Reaction Buffer	10.0
dNTP (10mM each)	2.0
HCV-specific probe HCVMGB2	0.5
Internal Control-probe YFPY	0.5
Forward primer XTF5	1.0
Reverse primer HCMgR2	1.5
Enzyme Mix	2.0
**TOTAL**	**30.0**
	**+ 20.0 RNA**

* all oligonucleotides are at 10µM concentration
* dNTP, reaction buffer and enzyme mix from the Qiagen One-Step RT-PCR Kit (or similar)
* RNA preparation with the Qiagen MinElute Spin Mini (or similar). If using a different extraction strategy, smaller amounts of RNA may be necessary to prevent from reaction inhibition.
* Follow general PCR precautions like pipetting on ice, calculating 10% pipetting reserve etc.

Oligonucleotide-Sequences:

XTF5	5'-GTGGCTCCATCTTAGCCCTAGT-3'
HCMgR2	5'-TGCGGCTCACGGACCTTT-3'
HCVMGB2	5'-FAM-CACGGCTAGCTGTG-MGBBHQ1-3'
YFPY	5'-VIC-ATCGTTCGTTGAGCGATTAGCAG-BHQ1-3'

Thermal cycling program (ABI 7700 or similar):

Step	Time	Temperature (°C)	Repetition
Reverse Transcription (RT)	10 min	55	
Enzyme activation and RT inactivation	15 min	95	
PCR	10 sec	94	X 45
	20 sec[a,b]	58	

[a]On ABI 7000, 7300 and 7500 platforms a minimum annealing-extension time of 33 seconds at 58°C may be obligatory

[b]Fluorescence readout at this step

Institute of Virology
University of Bonn Medical Centre
Sigmund Freud-Str. 25
53127 Bonn
Germany

For transfer of controls/standards:
Please contact via email: drexler@virology-bonn.de

Requirements prior to shipment:

1. Courier account number with confirmation of assumption of costs by recipient
2. Filled customs declaration forms as required in recipient's country

Shipment contents:
1 vial of plasmid template for quantification standard
1 vial of plasmid template for internal control

Please contact us separately if *in vitro*-transcribed RNA is requested

Institute of Virology
University of Bonn Medical Centre
Sigmund Freud-Str. 25
53127 Bonn
Germany

For transfer of controls/standards:
Please contact via email: drexler@virology-bonn.de
