# Supplementary material for: A Novel Diagnostic Target in the Hepatitis C Virus Genome
Source: PLoS Med. 2009 Feb 10;6(2):e1000031. doi: 10.1371/journal.pmed.1000031 (PMC2637920; doi:10.1371/journal.pmed.1000031)
Supplement: Supplementary file 3 [file pmed.1000031.sg001.pdf]

STARD Diagram for the evaluation of clinical sensitivity* (page 15)

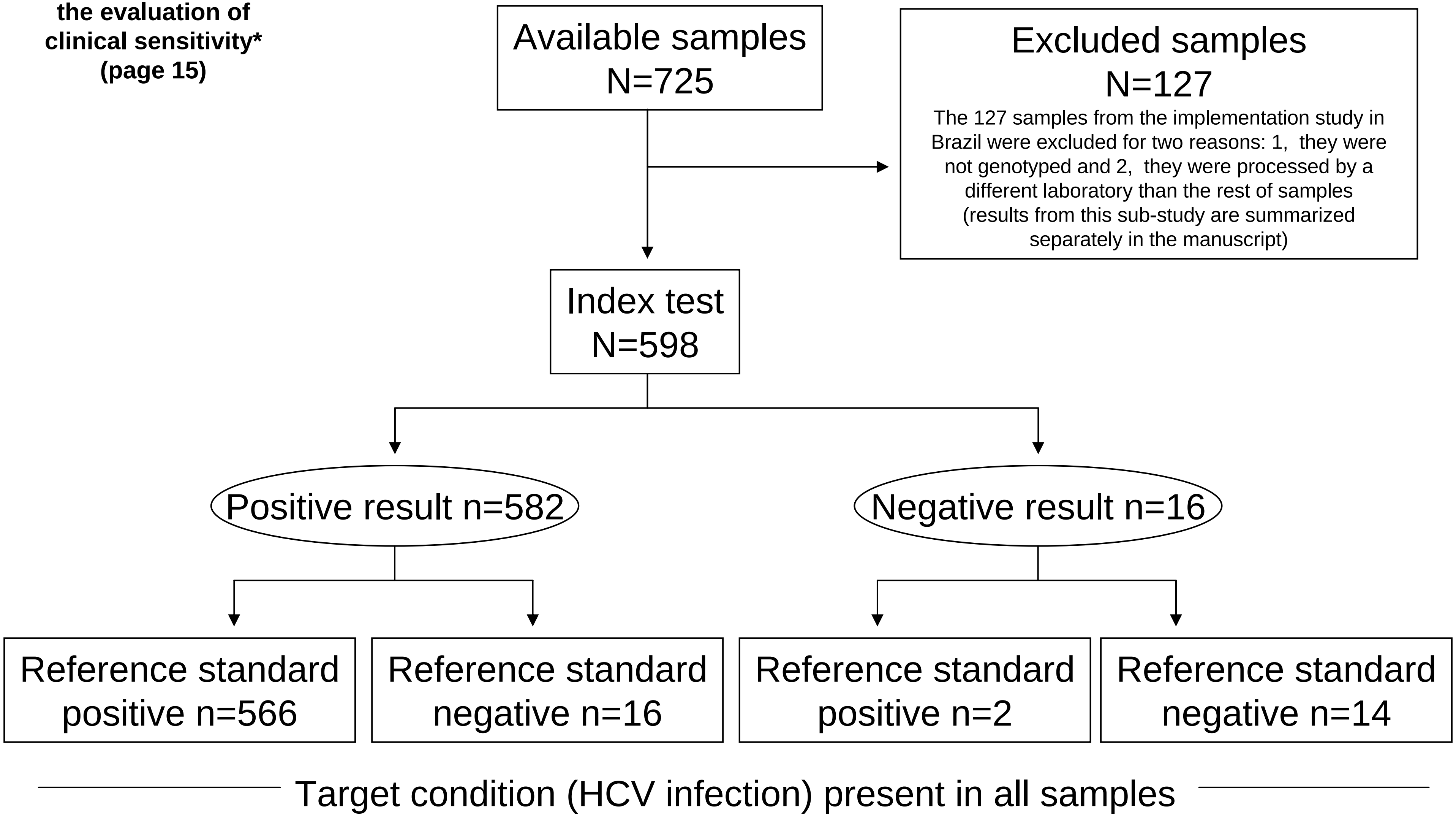

*In addition to clinical sensitivity we determined analytical sensitivities (limits of detection) for every genotype separately. This allows for a more general comparison of sensitivity with existing assays.
