# Supplementary material for: A Novel Diagnostic Target in the Hepatitis C Virus Genome
Source: PLoS Med. 2009 Feb 10;6(2):e1000031. doi: 10.1371/journal.pmed.1000031 (PMC2637920; doi:10.1371/journal.pmed.1000031)
Supplement: Supplementary file 4 [file pmed.1000031.sd001.doc]

**Résumé**

**Introduction:**

La détection et quantification de l'ARN du virus de l'hépatite C (VHC) sont primordiales dans les régimes diagnostiques et thérapeutiques. Tous les tests moléculaires ont pour cible la région 5’-non-codante du génome viral (en anglais, 5’-NCR), et tous montrent variation en ce qui concerne leur sensibilité et leur résultats de la charge virale entre les différents génotypes du VHC. Génotypes non-occidentaux du VHC sont sous-représentés dans les études d’évaluation. Une nouvelle cible diagnostique dans le génome du VHC pourrait faciliter une nouvelle génération de tests.
**Méthodes et résultats:**
Dans cette étude, il a été déterminé par séquençage *de novo* que l’élément 3’-X-tail, qui a été caractérise significativement plus tard que le reste du génome, est hautement conservé entre les génotypes du VHC. Pour prouver son utilité comme cible pour le diagnostic clinique moléculaire, un test prototype qualitatif et quantitatif a été développé et évalué dans une étude multicentrique sur un large panel complet de 725 échantillons cliniques de plasma, couvrant les génotypes 1-6 de VHC, de quatre continents (l'Allemagne , Royaume-Uni, Brésil, Afrique du Sud, Singapour). À notre connaissance, c'est le panel d'échantillons cliniques et de génotypes le plus complet et diversifié utilisé dans la validation d’un test de détection moléculaire (NAT) pour le VHC, à ce jour. La limite inférieure de détection (LOD) a été 18,4 UI/mL (intervalle de confiance 95%, 15,3-24,1 UI/mL), suggérant l'applicabilité de dépistage des donneurs de sang. La limite supérieure de détection dépasse 10^9^ UI/mL, ce qui facilite la surveillance de la charge virale dans une large gamme dynamique. Dans 598 échantillons de génotypes connus, quantifiés par Bayer VERSANT 3.0 bDNA, la charge virale basée sur la région X-tail a été très concordante avec bDNA pour tous les génotypes. Les coefficients de corrélation entre bDNA et X-tail NAT, pour les génotypes 1-6, ont été les suivants: 0.92, 0.85, 0.95, 0.91, 0.95 et 0.96, respectivement; les charges virales basées sur la région X-tail ont dévié de plus de 0,5 Log10 de la charge virale basée sur la région 5’-NCR que dans 12% des échantillons (écart maximal, 0,85 Log10). Le succès de l'introduction de NAT basée sur la région X-tail dans un laboratoire brésilien a confirmé la stabilité pratique et la robustesse du protocole. Le test a été mis en œuvre à coût bas par réaction (US$ 8,70 par échantillon), haut débit (2,5 h pour un maximum de 96 échantillons) et sans difficultés techniques.
**Conclusion:**
Cette étude indique une manière à améliorer fondamentalement la surveillance de la charge virale et le dépistage de l'infection par le VHC. Notre test prototype peut servir de modèle pour une nouvelle génération de tests de charge virale. En effet, cette étude fournit le premier protocole ouvert pour permettre la détection et la quantification du VHC au niveau de l'industrie dans les pays à ressources limitées.
