# Supplementary material for: A Novel Diagnostic Target in the Hepatitis C Virus Genome
Source: PLoS Med. 2009 Feb 10;6(2):e1000031. doi: 10.1371/journal.pmed.1000031 (PMC2637920; doi:10.1371/journal.pmed.1000031)
Supplement: Supplementary file 5 [file pmed.1000031.sd002.doc]

**Zusammenfassung**
**Einleitung:**
Der Nachweis und die Quantifizierung von HCV RNA sind integraler Bestandteil der Therapie der Hepatitis C. Alle molekularen Nachweisverfahren haben als Zielregion die virale 5'-noncoding Region (5’-NCR) und variierten hinsichtlich ihrer Sensitivität zwischen HCV Genotypen. Gerade solche Genotypen, die oft in Entwicklungs- und Schwellenländern vorkommen, sind in den Zulassungsstudien unterrepräsentiert. Eine alternative diagnostische Zielregion innerhalb des HCV-Genoms könnte eine neue Generation von Testen ermöglichen, die alle Genotypen zuverlässig quantifiziert.
**Methoden und Ergebnisse:**
In dieser Studie wurde durch *de novo* Sequenzierung gezeigt, dass das 3’-X-Tail Element, welches deutlich später als der Rest des Genoms charakterisiert wurde, hoch konserviert in allen HCV Genotypen ist. Um den klinischen Nutzen des X-tail als Zielregion für die molekulare Diagnostik zu belegen, wurde ein qualitativer und quantitativer Prototyptest entwickelt und multizentrisch an 725 Plasmaproben evaluiert. Die Probensammlung umfasste HCV Genotypen 1-6 aus vier Kontinenten (Deutschland, Großbritannien, Brasilien, Südafrika, Singapur). Unseres Wissens ist dies die umfassendste Studie hinsichtlich Genotypen, die bisher für die Validierung eines HCV-NAT Tests durchgeführt wurde. Die untere Nachweisgrenze des Prototyptests (LOD) lag bei 18,4 IE/mL (95% Konfidenzintervall, 15,3-24,1 IE/mL), wodurch auch eine Anwendbarkeit im Blutspenderscreening gegeben wäre. Die obere LOD überschritt 10^9^ IE/mL, was einer Viruslastmessung mit weitem dynamischen Messbereich ermöglicht. Bei 598 Proben aller Genotypen stimmte die X-Tail-basierte Viruslast in hohem Maße mit den Ergebnissen der zuverlässigen bDNA Methode (Bayer VERSANT 3.0) überein. Korrelationskoeffizienten zwischen bDNA und X-Tail Tests waren für die Genotypen 1-6 jeweils: 0,92, 0,85, 0,95, 0,91, 0,95 und 0,96. Die X-Tail-basierten Viruslasten wichen nur bei 12% aller Proben um mehr als 0,5 Log10 von 5’-NCR -basierten Viruslasten ab (maximale Abweichung 0,85 Log10). Die erfolgreiche Einführung von X-Tail-NAT in einem brasilianischen Labor bestätigt die praktische Stabilität und Robustheit des X-Tail basierten Protokolls. Der Test wurde bei niedrigen Reaktionskosten (8,70 US $ pro Probe), hohem Durchsatz (2,5 h für bis zu 96 Proben) und ohne technische Schwierigkeiten in einem brasilianischen Labor etabliert.
**Schlussfolgerungen:**
Diese Studie zeigt einen Weg auf, Nachweisverfahren zum qualitativen und quantitativen HCV-Nachweis grundlegend zu verbessern. Der vorgestellte Prototyptest kann als Vorlage für eine neue Generation von HCV-Testen dienen. Als Nebeneffekt stellt diese Studie das erste offene zugängliche Protokoll zur Verfügung, welches einen HCV-Nachweis nach derzeit gültigen Industriestandards gestattet und in Ländern mit begrenzten finanziellen Ressourcen einsetzbar ist.
