# Supplementary material for: A Novel Diagnostic Target in the Hepatitis C Virus Genome
Source: PLoS Med. 2009 Feb 10;6(2):e1000031. doi: 10.1371/journal.pmed.1000031 (PMC2637920; doi:10.1371/journal.pmed.1000031)
Supplement: Supplementary file 6 [file pmed.1000031.sd003.doc]

**Resumo**

**Introdução:**

A detecção e quantificação do RNA do vírus da hepatite C (VHC) é primordial em esquemas diagnósticos e terapêuticos. Todos os ensaios moleculares têm como alvo a região 5’-não traduzida do genoma viral (do inglês 5’-NCR) e mostram variação de sensibilidade e na determinação da carga viral entre os diferentes genótipos do VHC. Genótipos não-ocidentais do VHC têm sido sub-representados nos estudos avaliativos. Um novo alvo diagnóstico no genoma do VHC poderá constituir uma alternativa para uma nova geração de ensaios.

**Métodos e resultados:**
Neste estudo, foi determinado por seqüenciamento *de-novo* que a extremidade 3’ do genoma do VHC denominada 3’-X-tail, , é altamente conservada entre os genótipos do VHC. Para demonstrar sua utilidade clínica como alvo diagnóstico molecular, um teste protótipo qualitativo e quantitativo foi desenvolvido e avaliado em estudo multicêntrico sobre um amplo e completo painel de 725 amostras clínicas de plasma, abrangendo genótipos 1-6 do VHC, de quatro continentes (Alemanha, Reino Unido, Brasil, África do Sul, Singapura). Trata-se do mais diversificado e abrangente painel de amostras clínicas e genótipos utilizado na validação de um teste de detecção do VHC por métodos moleculares (NAT), até a presente data. O limite inferior de detecção (LOD) foi de 18,4 UI/mL (95% intervalo de confiança, 15,3-24,1 UI/mL), sugerindo aplicabilidade na triagem de doadores de sangue. O limíte superior de detecção ultrapassou 10e9 UI/mL, facilitando o monitoramento da carga viral num amplo e dinâmico intervalo. Em 598 amostras genotipadas, quantificadas pelo kit Bayer VERSANT 3.0 bDNA, as cargas virais baseadas na região X-tail foram altamente concordantes com bDNA para todos os genótipos. Coeficientes de correlação entre bDNA e X-tail NAT, para genótipos 1-6, foram: 0,92, 0,85, 0,95, 0,91, 0,95 e 0,96, respectivamente; cargas virais baseadas na região X-tail desviaram por mais de 0,5 Log10 de cargas virais baseadas na região 5’-NCR em apenas 12% das amostras (desvio máximo, 0,85 Log10). A introdução bem sucedida de NAT baseado na região X-tail em um laboratório Brasileiro confirmou a estabilidade prática e a robustez do protocolo. O ensaio foi implementado a baixo custo por reação (8,70 US$ por amostra), alta rotatividade (2,5 h para até 96 amostras) e sem dificuldades técnicas.

**Conclusão:**
Este estudo indica um caminho para melhorar fundamentalmente o monitoramento da carga viral e rastreamento de infecção pelo VHC. A presente proposta pode servir como um modelo para uma nova geração de testes, constituindo o primeiro protocolo aberto para permitir a detecção e quantificação do VHC em larga escala em locais de recursos limitados.
