# Supplementary material for: A Novel Diagnostic Target in the Hepatitis C Virus Genome
Source: PLoS Med. 2009 Feb 10;6(2):e1000031. doi: 10.1371/journal.pmed.1000031 (PMC2637920; doi:10.1371/journal.pmed.1000031)
Supplement: Supplementary file 7 [file pmed.1000031.sd004.doc]

**Resumen**

**Introducción:**
La detección y cuantificación del ARN del virus de la hepatitis C (VHC) es parte integrante de los regímenes diagnósticos y terapéuticos. Todas las pruebas moleculares se han diseñado para la región 5’-no codificante del genoma viral (del inglés, 5’-NCR), y todas presentan variación de la sensibilidad y de los resultados de carga viral entre los diferentes genotipos del VHC. Los genotipos no occidentales del VHC no están suficientemente representados en los estudios de evaluación. Un blanco diagnóstico alternativo en el genoma del VHC podría facilitar una nueva generación de pruebas.
**Métodos y resultados:**
En este estudio, fue determinado mediante secuenciación *de novo* que el elemento 3’-X-tail, caracterizado significativamente más tarde que el resto del genoma, tiene un alto grado de conservación entre los genotipos del VHC. Para probar su utilidad como blanco diagnóstico clínico molecular se desarrolló y evaluó una prueba prototipo cualitativa y cuantitativa en un estudio multicéntrico sobre un amplio y completo panel de 725 muestras clínicas de plasma, que abarca genotipos 1-6 de VHC de cuatro continentes (Alemania, Reino Unido, Brasil, Sudáfrica, Singapur). Para nuestro conocimiento, este es el más completo y variado panel de muestras clínicas y genotipos utilizado en la validación de una prueba de detección molecular (NAT) para el VHC hasta la fecha. El límite inferior de detección (LOD) fue 18,4 UI/mL (intervalo de confianza 95%, 15,3-24,1 IU/mL), lo que sugiere su aplicabilidad en la selección de donantes de sangre. El límite superior de detección superó 10^9^ UI/mL, facilitando la monitorización de la carga viral dentro de una gama dinámica muy amplia. En 598 muestras genotipadas, cuantificadas por Bayer Versant 3.0 bDNA, las cargas virales basadas en la región X-tail fueron muy concordantes con bDNA para todos los genotipos. Los coeficientes de correlación entre bDNA y X-tail NAT, para los genotipos 1-6, fueron: 0,92, 0,85, 0,95, 0,91, 0,95 y 0,96, respectivamente; la carga viral basada en la región X-tail se desvió en más de 0,5 Log10 de la carga viral basada en la región 5'-NCR en sólo el 12% de las muestras (desviación máxima, 0,85 Log10). El éxito de la introducción de X-tail NAT en un laboratorio de Brasil confirmó la estabilidad práctica y la solidez del protocolo basado en la región X-tail. La prueba se aplicó a un coste bajo por reacción (8,70 US$ por muestra), con un alto volumen de muestras procesadas (2,5 h hasta un máximo de 96 muestras) y sin dificultades técnicas.
**Conclusión:**
Este estudio indica una forma de mejorar fundamentalmente la monitorización de la carga viral y la detección de la infección por el VHC. Nuestra prueba prototipo puede servir como modelo para una nueva generación de pruebas de carga viral. Como efecto secundario, este estudio proporciona el primer protocolo abierto que permite la detección y cuantificación de VHC a nivel industrial en entornos con recursos limitados.
