# Supplementary material for: A Novel Diagnostic Target in the Hepatitis C Virus Genome
Source: PLoS Med. 2009 Feb 10;6(2):e1000031. doi: 10.1371/journal.pmed.1000031 (PMC2637920; doi:10.1371/journal.pmed.1000031)
Supplement: Supplementary file 8 [file pmed.1000031.sd006.doc]

**Drexler et al., "A novel diagnostic target in the hepatitis C virus genome"**

**Supplemental Text S2**

**Technical details and evaluation of the X-tail RT-PCR assay**

**Ultrasensitive extraction of HCV RNA from plasma**

RNA was extracted on the automated QiaCube instrument (Qiagen, Hilden, Germany), using the Qiagen MinElute Spin kit. Alternatively the Qiagen MinElute Vacuum protocol was used. Plasma input volume was 200 µL in the first procedure and 500 µL in the latter. Elution volume was 25 µL in both.

**Cloning, *in-vitro* transcription and nucleic acid sequencing**

cDNA from HCV reference samples was TA-cloned in pCR 2.1 (Invitrogen, Karlsruhe, Germany). Plasmid inserts were transcribed into RNA with the MegaScript T7 *in-vitro* transcription kit (Ambion, Austin, TX, USA), followed by purification, quantification and sequencing as described [1,2].

**Quantification standard**

An incomplete copy of the HCV 1a X-tail region was transferred from cloned cDNA into MS2 coliphage (Ambion, Houston, TX, USA). The resulting preparation contained 10e14 particles of target RNA in non-infectious and environmentally stable form [3]. For calibration, Armored RNA was diluted in Fresh Frozen Plasma (FFP) to concentrations of 1 to 6 Log10 above the X-tail RT-PCR detection limit. Four replicates of each concentration were processed and amplified by real-time PCR, along with 4,000, 800, 160 and 32 IU/mL of WHO HCV international standard. The latter dilution series was defined as standard curve samples in real-time PCR. The concentration of the Armored RNA working solution was projected from the determined viral loads at each concentration step. The quantified Armored RNA was then used for setting up quantification standard curves in each assay. Of note, calibration with the WHO standard resulted in a systematic overestimation of viral loads versus the bDNA assay by a factor of 1.9. Although this deviation would be clinically irrelevant and in concordance with the literature, an alternative calibration was done against bDNA. To this end the calibration experiment used bDNA-quantified plasma samples instead of diluted WHO standard. In this study we used the calibration against bDNA.

**Internal Control**

Primers HcvICS2 (5-TGGTGGCTCCATCTTAGCCCTAGTATCGTTCGTTGAGCGATTAGCAG) and HcvICAs2: (5-TGCGGCTCACGGACCTTTCTGCTAATCGCTCAACGAACGAT) were extended without virus template by 40 PCR cycles. The resulting 65 bp dsDNA represented an exact copy of the X-tail RT-PCR amplicon with an exchanged probe binding site. It was TA-cloned and *in-vitro* transcribed with the MegaScript kit. For *in-vitro* transcription the template cDNA was amplified from plasmid with vector-specific primers to generate an 850 nt extension downstream of the insert sequence, ensuring more efficient purification with silica-membrane based extraction methods later on [2]. To enhance stability of the transcript, the UTP nucleotide in *in-vitro* transcription was substituted by 2’-iodized UTP (Biozym, Hessisch Oldendorf, Germany).The resulting synthetic RNA was quantified photometrically. The transcript copy number to be added as an internal control (IC) without affecting PCR sensitivity for HCV was adjusted in limiting dilution experiments, always using 5 replicate tests per concentration. With up to 90 copies of IC added, a calculated 7.5 IU of HCV 1a RNA were detectable in the same reaction, in 5 of 5 parallel assays, without noticeable suppression of amplification. Only from 2,750 copies per reaction onwards, HCV amplification (7.5 IU) was totally suppressed. Between 90 and 2,750 copies partial suppression occurred. Fifty copies of IC per reaction were chosen as the working concentration. It was confirmed in further experiments that 27, 9 and 3 IU of other HCV genotypes were also not suppressed at this IC concentration.

**PCR interpretation**

Fluorescence was read out at the 58ºC step of the final segment of the cycling program. The baseline area of fluorescence signal was routinely defined as cycles 3 to 15. Standard curves were used as automatically obtained from the operation software of the real-time PCR system. A uniform threshold line was set manually for all samples. For those reactions that did not yield FAM amplification signals, the VIC amplification signal was inspected. In case the signal was negative or delayed against the average VIC signal in the negative plasma controls by two cycles or more, the respective sample was considered invalid due to PCR inhibition. Such samples were re-processed and re-tested at their original concentrations, as well as after dilution 1:10 in FFP from HCV-negative donors.

**Determination of lower detection limit and intra- / inter-assay variability**

The WHO international HCV RNA standard was diluted in fresh frozen plasma to 54, 18, 6 and 2 IU/ml plasma. Five replicate samples of each concentration were processed as for patient samples and tested. Sample counts and fractions of positive samples at each concentration were used for probit regression analysis with the Statgraphics software package (Manugistics, Dresden, Germany). At an extraction input of 500 µL plasma, the overall dose-response model including all genotypes showed an LOD of 18.4 IU/mL that was detected with >95% probability (95% confidence interval (CI), 15.3-24.1 IU/ml) (Figure S4). Because 500 µL of plasma were not always available for testing, e.g., in stored samples or in samples from children, the protocol was also evaluated with a reduced input volume of 200 µL for the clinical evaluation study. With this version the average LOD was 46 IU/mL (data not shown). The LODs with the two different extraction protocols (18.4 IU/ml for 500 µL and 46 IU/ml for 200 µL plasma input) indicated no or little underlying inhibitory effect contributed by high plasma input.

The intra-assay variability was assessed by testing a genotype 1 sample in 8 replicates in the same experiment. Mean viral load was 7,245,000 IU/mL with a coefficient of variation (CV) of 5.76% and a standard deviation (SD) of 0.03Log10. To evaluate the inter-assay variability, plasma samples containing 189,000, 18,900 and 1,890 IU/ml of armored RNA, respectively, were tested on five different days. CT values were transformed to IU/ml based on the slope of the quantification curve. Coefficients of variation were 9.21, 18.42 and 6.11 %, respectively, from highest to lowest concentrations. Mean determined quantification results for the three samples were 169,989, 19,763 and 2,102 IU/ml with standard deviations of 0.03, 0.09 and 0.08 Log10, respectively.

For all genotypes, a slight overquantification against bDNA was observed in samples with viral loads close to the upper detection limit of bDNA. It is not clear if this represents a ceiling effect in the bDNA assay or a phenomenon inherent to X-tail NAT. However, a recent study showed similar data for genotypes 1-3 when comparing the new Roche Cobas TaqMan assay and bDNA [4]. Studies using alternative gold standards are required to address this issue in more detail.

**References**

1. Drosten C, Seifried E, Roth WK (2001) TaqMan 5'-nuclease human immunodeficiency virus type 1 PCR assay with phage-packaged competitive internal control for high-throughput blood donor screening. J Clin Microbiol 39: 4302-4308.

2. Drosten C, Weber M, Seifried E, Roth WK (2000) Evaluation of a new PCR assay with competitive internal control sequence for blood donor screening. Transfusion 40: 718-724.

3. Walkerpeach CR, Pasloske BL (2004) DNA bacteriophage as controls for clinical viral testing. Clin Chem 50: 1970-1971.

4. Chevaliez S, Bouvier-Alias M, Brillet R, Pawlotsky JM (2007) Overestimation and underestimation of hepatitis C virus RNA levels in a widely used real-time polymerase chain reaction-based method. Hepatology 46: 22-31.

5. Lee SC, Antony A, Lee N, Leibow J, Yang JQ, et al. (2000) Improved version 2.0 qualitative and quantitative AMPLICOR reverse transcription-PCR tests for hepatitis C virus RNA: calibration to international units, enhanced genotype reactivity, and performance characteristics. J Clin Microbiol 38: 4171-4179.

6. Kolykhalov AA, Feinstone SM, Rice CM (1996) Identification of a highly conserved sequence element at the 3' terminus of hepatitis C virus genome RNA. J Virol 70: 3363-3371.

**Supplemental figures**

**Figure S2. Alignment showing mismatches at Roche Amplicor oligonucleotide binding sites**

Nucleotide alignment of all the 5’-NCR sequences of all 60 members of the LANL genotype reference panel as available in August 2008 (<http://hcv.lanl.gov/content/sequence/NEWALIGN/align.html>). All sequences are identified by their genotypes, sample IDs and accession numbers as provided by LANL. Dots represent identities; capital letters show deviations from the head sequence. Dashes indicate missing sequence data in the LANL reference panel. Roche Amplicor version 2.0 oligonucleotides were retrieved from [5]. The antisense primer is depicted as its codogenic, i.e., reverse complement, strand.

**Figure S3.** **Alignment showing mismatches at X-tail RT-PCR oligonucleotide binding sites**

Nucleotide alignment of all 140 HCV X-tail sequences as available in January 2007 from online domains and the 10 members of the obtained HCV genotype reference panel. All GenBank sequences are identified by their accession numbers and genotypes as given by the publisher, when available. Only GenBank sequences that covered the entire PCR amplicon were included. Dots represent identities, capital letters a nucleotide mismatch to the binding region of the oligonucleotide depicted on top of the alignment. Gaps between oligonucleotides were introduced manually for better distinction. The antisense primer is depicted as its codogenic, i.e., reverse complement, strand.

Only one nucleotide mismatch (T-T) was present at the 5’ end of the antisense primer R2 in reference genotype 3a. This mismatch appeared in 24 additional GenBank sequences and was highly unlikely to interfere with PCR performance, as were all of the remaining occasional mismatches in a few GenBank sequences (Figure 3). Most important, only one single GenBank sequence showed one single mismatch at the probe binding site (5’-9 position, G-G). Marked with asterisk (* ) is sequence AF009077 which belongs to one of the samples used in the original description of the X-tail [6]. This sample was later found to contain an HCV genotype 4, but is still classified as 1a in LANL.

**Figure S4. Probit regression analysis to determine the X-tail RT-PCR limit of detection**

Probability of detection (y axis) is plotted against RNA concentration in 50 parallel test samples per data point (x axis). The plot depicts the observed proportion of positive results in parallel experiments (■), as well as the derived predicted proportion of positive results at a given input concentration of RNA. The solid line is the prediction; the dashed lines are the 95% confidence limits for the prediction.
